# Building a Chatbot in a Pandemic

**DOI:** 10.2196/42960

**Published:** 2023-10-10

**Authors:** Kimberly Rambaud, Simon van Woerden, Leonardo Palumbo, Cristiana Salvi, Catherine Smallwood, Gerald Rockenschaub, Michail Okoliyski, Lora Marinova, Galina Fomaidi, Malika Djalalova, Nabiha Faruqui, Viviane Melo Bianco, Mario Mosquera, Ivaylo Spasov, Yekaterina Totskaya

**Affiliations:** 1 World Health Organization Regional Office for Europe Copenhagen Denmark; 2 WHO Country Office Bulgaria Sofia Bulgaria; 3 WHO Country Office Uzbekistan Tashkent Uzbekistan; 4 United Nations Children's Fund, Europe and Central Asia Regional Office Geneva Switzerland; 5 UNICEF Country Office Bulgaria Sofia Bulgaria; 6 UNICEF Country Office Uzbekistan Tashkent Uzbekistan

**Keywords:** COVID-19, chatbots, evidence-based communication channels, conversational agent, user-centered, health promotion, digital health intervention, online health information, digital health tool, health communication

## Abstract

Easy access to evidence-based information on COVID-19 within an infodemic has been a challenging task. Chatbots have been introduced in times of emergency, when human resources are stretched thin and individuals need a user-centered resource. The World Health Organization Regional Office for Europe and UNICEF (United Nations Children's Fund) Europe and Central Asia came together to build a chatbot, HealthBuddy+, to assist country populations in the region to access accurate COVID-19 information in the local languages, adapted to the country context. Working in close collaboration with thematic technical experts, colleagues and counterparts at the country level allowed the project to be tailored to a diverse range of subtopics. To ensure that HealthBuddy+ was relevant and useful in countries across the region, the 2 regional offices worked closely with their counterparts in country offices, which were essential in partnering with national authorities, engaging communities, promoting the tool, and identifying the most relevant communication channels in which to embed HealthBuddy+. Over the past 2 years, the project has expanded from a web-based chatbot in 7 languages to a multistream, multifunction chatbot available in 16 regional languages, and HealthBuddy+ continues to expand and adjust to meet emerging health emergency needs.

## Introduction

The COVID-19 pandemic has greatly emphasized the importance of risk communication and community engagement (RCCE) for behavioral interventions for community protection. Giving people rapid access to easy-to-understand, reliable health information and advice is one of the most effective actions that emergency health responders can take—especially when little is known, and other countermeasures are not available. An absence of evidence-based information provided immediately to the public, even in the context of significant uncertainty, can leave room for misinformation and disinformation to fill the void.

Equally important are the relationships between groups of at-risk people and the health authorities that aim to protect them. Trust in those relationships has been shown to be one of the best predictors of success, and feedback from at-risk and affected communities is a keystone of a community-centered and effective emergency response.

In January 2020, following the declaration of the novel coronavirus as a public health emergency of international concern, the World Health Organization (WHO) Regional Office for Europe and UNICEF (United Nations Children's Fund) Europe and Central Asia Regional Office forged a partnership to rapidly develop a chatbot tool, HealthBuddy+, which would allow the 2 agencies to jointly share up-to-date, evidence-based COVID-19 information directly with the public via national authorities and other trusted national websites [1]. Considering the COVID-19 pandemic’s unique requirement for individual action, such as physical distancing, RCCE and information-sharing mechanisms are needed for rapid and broad scalability to reach target demographics [2]. Chatbots have been identified as a potentially efficient solution to complement the provision of health information, particularly when infrastructure and human resources are strained [3]. To deliver essential health information during a public health emergency, a triage approach involving a range of stakeholders can be taken to ensure that messages are aligned and adapted to local realities and needs.

The first iteration of HealthBuddy+ was launched in May 2020 across the WHO European Region [1] and aimed to translate the swiftly developing, complex technical guidance and recommendations on COVID-19 into user-friendly, accessible messaging from a health literacy and harm- and risk-reduction perspective.

The HealthBuddy+ chatbot was designed with an emphasis on equipping users with information on the disease and its prevention and care, and described as an information dissemination tool [2]. To disseminate COVID-19 information more locally, the content was translated into 7 languages initially, then expanded. Users were thus able to access easy-to-understand COVID-19 information and advice in their own languages. To better equip users with important information on prevention and protection, the content focused on helping them distinguish myth from fact and access key national sources of information, such as on testing and quarantine or isolation requirements.

With rumors circulating rapidly in the early days of the pandemic, the tool was also imagined as a trustworthy portal to fact-check rumors circulating about the virus. The infodemic that coincided with the pandemic [4] confirmed the urgent need to debunk rumors quickly, which presented the region with the challenge to scale up the dissemination of correct information and an opportunity to do so via HealthBuddy+. Following its success and the demand from the WHO and UNICEF country offices and their national partners, the chatbot was expanded to 16 languages and a multifunction mobile app introduced, which included a rumor-reporting tool as well as a user poll.

The aim of this viewpoint article is to reflect on the past 2 years of HealthBuddy+, with all the challenges, innovations, and lessons learned in deploying a chatbot during the COVID-19 pandemic.

## The Technology

Healthbuddy+ is an artificially intelligent conversational chatbot based on natural language processing principles. The chatbot is built on RapidPro, an open-source platform that allows users to design, build, and scale up mobile-based services. The advantage of using RapidPro in the HealthBuddy+ project is that it allows access to multiple languages and the ability to connect with external systems and social media messaging platforms through the custom-built RapidPro application programming interface.

**Figure 1:**
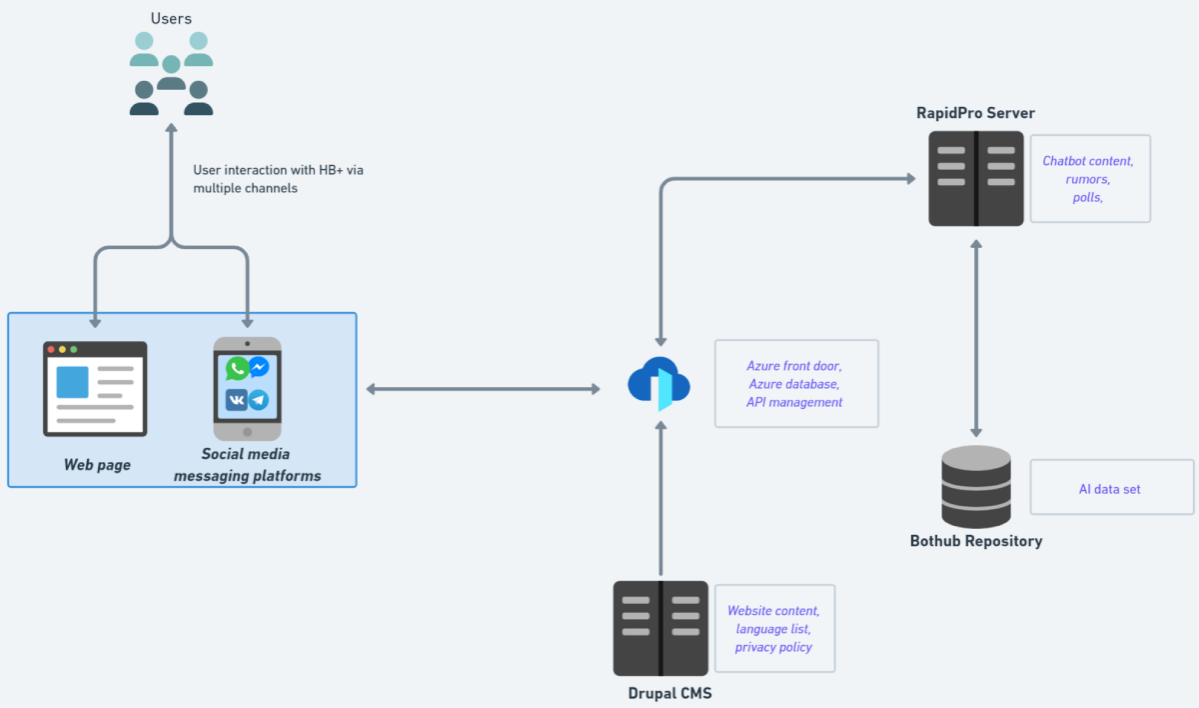
The technical architecture of HealthBuddy+. AI: artificial intelligence; API: application programming interface; CMS: content management system; HB+: HealthBuddy+.

**Figure 2:**
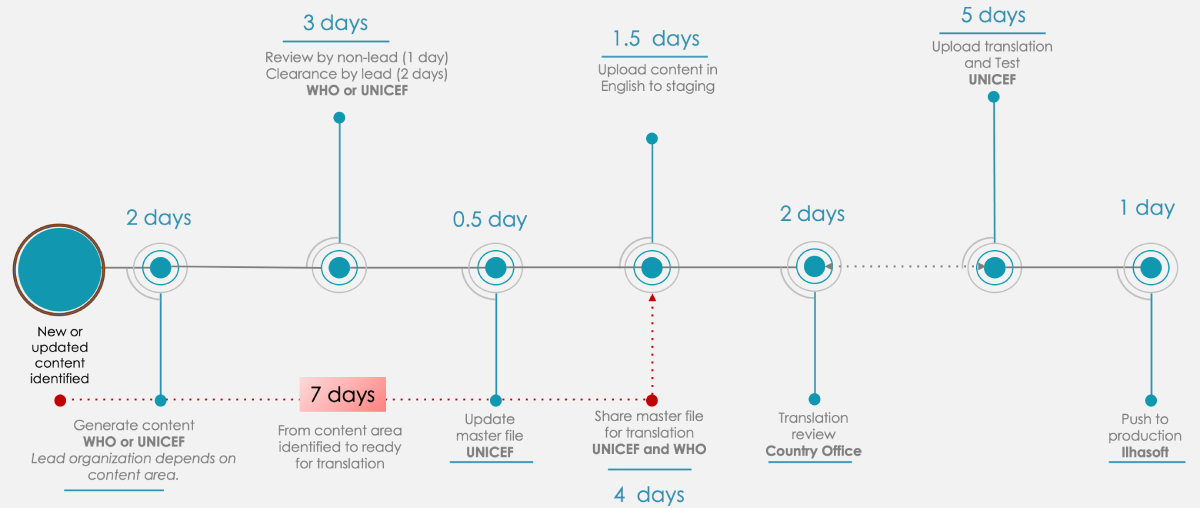
Pipeline for content updates from translation to live. UNICEF: United Nations Children's Fund; WHO: World Health Organization.

HealthBuddy+ is also connected via Bothub, which is another open-source platform. Bothub allows intelligence building by providing services such as intent classification, response retrieval, and entity extraction. It also provides the additional features of training intelligence in multiple languages. The technical architecture of HealthBuddy+ and the pipeline for content updates is shown in Figure 1 and Figure 2, respectively.

From the project experience, lessons learned were identified and assessed on an ongoing basis via the regional team (weekly) and country office focal points (monthly). These monthly meetings allowed focal points from across the region to present challenges and feedback at the country level, be it new content suggestions, ideas for promotion, or difficulties with the technology. The feedback was discussed among the regional team who took action as needed. Updates based on country feedback were then shared in the following month’s meeting. In this way, the experiences gained from project implementers at the country and regional levels were used to further refine HealthBuddy+.

The following sections explore the lessons learned and innovations made within the following 3 main pillars of the project: project management, country partnership, and content and translation—each with their unique challenges—as well as the frequent adjustments made by the HealthBuddy+ team to keep pace with the unfolding pandemic.

## Project Management

The regional project team consisted of 2 project managers (1 from each agency) and the following 3 focal points: content development and translation, technology, and promotion. The WHO Regional Office for Europe with an already mobilized COVID-19 emergency response team (COVID-19 Incident Management Support Team) was able to pull rapidly evolving technical information on COVID-19 to build the content base of the chatbot as well as initiate contact with focal points in country offices for support with translation and for in-country launch. Drawing from previous experience of using a chatbot and data collection platforms such as BotHub (discussed below in the Technology section) and U-Report [5] as well as standing partnerships with the technology sector, UNICEF Europe and Central Asia Regional Office was well positioned to bring the technical know-how of developing chatbots and engaging partners to build the platform and technology behind HealthBuddy+. Combining the strengths of the 2 organizations allowed for the rapid launch of the chatbot.

The concept was validated with polls, which suggested that citizens in the WHO European Region generally trusted the WHO and UNICEF as organizations that provide credible and trustworthy advice on how to protect oneself from COVID-19. At the same time, the data showed that many people used their Ministry of Health web pages to access the latest information and advice. This was achieved by embedding the chatbot on these trusted channels in strong partnership with member states and partners (including the health sector but also national mainstream media and civil society organizations) and funneling trustworthy health information to the user. In this instance, HealthBuddy+ exemplified the core RCCE concepts of building and maintaining trust by “using channels your audience uses and trusts” to distribute accessible and essential COVID-19 information [6].

## Country Partnership and Innovation

**Figure 3:**
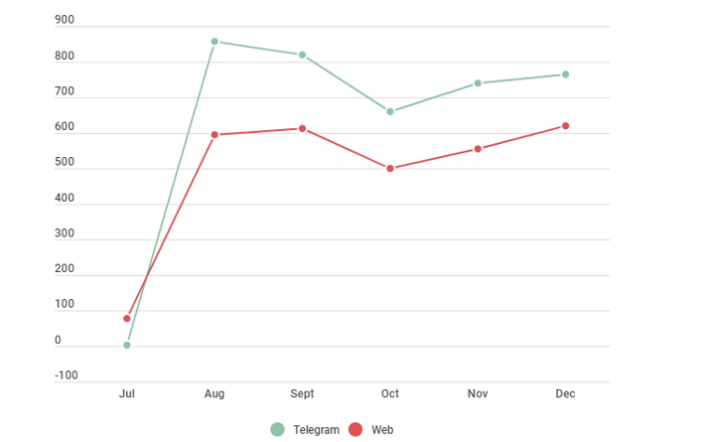
Country example using trusted channels: Uzbekistan was the first partnering country to embed HealthBuddy+ into a popular messaging platform (Telegram), which it did in August 2021. The graph illustrates how incorporating the chatbot where citizens already communicate can increase the number of users overall.

The core objective of working in partnership with colleagues in country offices was to ensure that HealthBuddy+ was adapted to reach citizens via effective channels of communication. To meet this objective, communication focal points in the WHO and UNICEF offices were identified in 15 countries and territories (See Textbox 1 for full list). Their role included, but was not limited to, translation support, identifying local and national resources to link users to the chatbot, promotion and campaign development, and advocacy. When identifying channels within which to embed the chatbot, it was paramount to work closely with country focal points to understand which messaging channels were the most relevant. Researchers have previously recommended the popular and highly used messaging channels (eg, WhatsApp, Viber, Telegram, Facebook Messenger, etc) as possible hosts for health chatbots, as opposed to isolated websites or applications, which were of less interest to users over time [7]. With the technology evolving and HealthBuddy+ being adopted rapidly in multiple countries, the need arose to expand the reach and meet users where they are already communicating. This led to the integration of HealthBuddy+ with the abovementioned social media messaging platforms. The number of users via these platforms as of January 24 2023, is shown in Table 1, and a country example in Figure 3.

**Figure 4:**
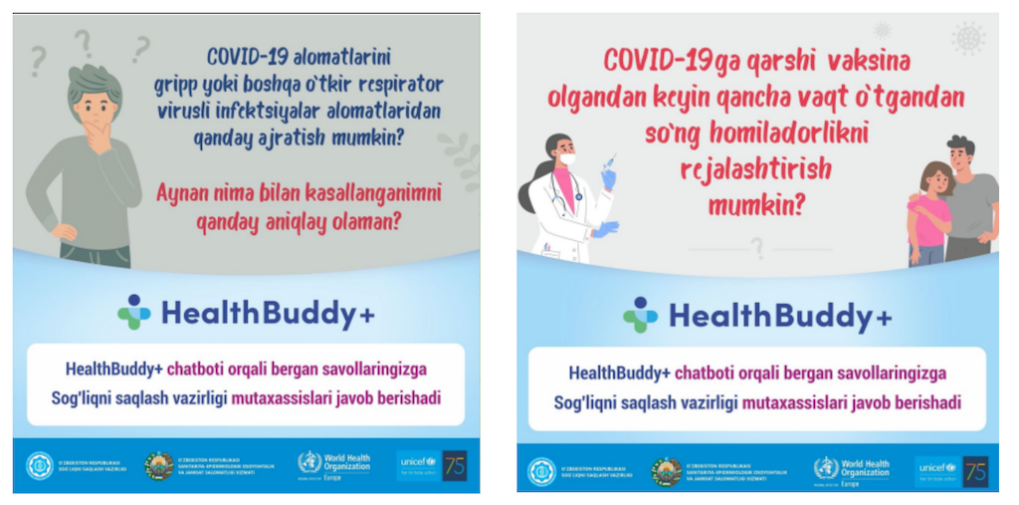
Translation of left-hand panel: How to distinguish the symptoms of COVID-19 from the symptoms of the flu and other respiratory diseases? How can I find out what I have been infected with? Translation of right-hand panel: How long should I wait after full vaccination before planning a pregnancy?

Once HealthBuddy+ had been launched in several countries, user questions began to pour in, which were uploaded into a backend system, which was log-in secured, whereby only selected project members had access. The user questions inquired on topics ranging from symptom recognition to local public health and safety measures, and transmission methods, as well as local mis- and disinformation (see Textbox 2 and Figure 4 for country examples). As requested, country focal points could receive a download of their language’s anonymized questions to better understand important information gaps as well as circulating rumors. There was an abundance of questions at the start of the launch, which declined over time as the bot’s intelligence grew based on the range and frequency of the questions asked; in short, the more questions, the smarter the bot.

**Full list of participating countries and territories.**

Armenia, Belarus, Bulgaria, Greece, Hungary, Kazakhstan, Kyrgyzstan, Kosovo (all references to Kosovo in this document should be understood to be in the context of the 1999 United Nations Security Council resolution 1244), North Macedonia, Poland, Romania, Russian Federation, Türkyie, Ukraine, and Uzbekistan.

**Table 1:** Number of users via different channels (as of January 24, 2023).

Channel	Number of users
Web (including website and all embeds in country websites)	3,771,918
Viber	521,874
Mobile app	135,776
Telegram	69,184
Facebook Messenger	2872
WhatsApp	905

**Country examples: filling information voids.**

In Bulgaria, focal points from the World Health Organization (WHO) and UNICEF capitalized on the translation of user questions to turn these into an opportunity to directly respond by:

- Addressing the main questions and debunking reoccurring rumors during the public launch event of HealthBuddy+.
- Using HealthBuddy+ user questions for wider risk communication and community engagement efforts, notably by summarizing and using these questions as a point of engagement in public appearances, such as in national television interviews, where the questions were addressed and viewers were directed to guidance from the WHO tailored to the Bulgarian context.
- Analyzing user questions to highlight major COVID-19 themes of interest in the country, which were addressed in COVID-19 prevention webinars.

Focal points in Uzbekistan translated user questions so they could be addressed at the regional level and analyzed them to produce social media tiles to fill information voids. Some examples of these tiles can be seen in Figure 4.

Maintaining such a massive content repository in over 20 languages posed a significant challenge on its own; the constantly evolving pandemic meant that the content had to be updated frequently, which also meant that the intelligence had to be upgraded simultaneously. Working with the regional offices, country offices came up with creative ideas on how to respond to user questions while the bot’s intelligence was growing. The user questions also presented the team with an opportunity to go beyond one-way information provision and understand user experience and what users wanted to know (ie, lay use of technical terminology, types of questions, fears and concerns, choice of words, and ways of speaking) and then proactively aim to fill voids based on these insights.

## Content and Translation

The approach to the content development process has evolved significantly from the project start where little to nothing was known about COVID-19. The approach shifted from focusing on whatever evidence-based information was available to a content development process that incorporated more user-oriented questions. The content base grew from roughly 20 questions and answers to over 200 in the span of a year and a half. The two primary functions of the content are as follows:

- Questions and answers, either via the question menu or a user-typed question
- Referral pathway to local resources (hotlines, local websites, etc).

An inherent challenge to HealthBuddy+’s content development was incorporating content based on global guidance and making it relevant and useful throughout the diverse region of 53 member states. To remedy this challenge, the team placed the user at the center and informed their understanding of the user through several channels, including the following:

1. Broader social listening insights via regular social and media listening and behavioral and cultural insights.
2. User data, highlighted while filling the information voids mentioned above.
3. Insights from country focal points, who regularly shared the hot topics circulating in their localities.

As the technical guidance and recommendations evolved, for instance, with the introduction of vaccines, the team was able to share the latest trustworthy and evidence-based information, while simultaneously collecting listening data via the user poll.

As HealthBuddy+ strived from the start to be an information resource that is accessible to audiences across the linguistically diverse WHO European region, translating the content into 16 languages was a key component of the project’s uniqueness. This, however, was not without its challenges; initially translations were not centralized, which meant some were translated locally and others via the regional team’s translation service, which made it difficult to track the progress and time updates. Regional-level translation was fast and competitively priced; however, to ensure technical terms and nuanced phrasing was translated accurately, the translations needed to be verified by the focal points, adding an additional layer to their work and an additional step to the content update process.

## Discussion

### Principal Findings

The COVID-19 pandemic rapidly shifted the use of digital tools, including conversational RCCE tools, from an innovative possibility to an urgent need [4].

Literature on the use of chatbots in health has been rising steadily [8,9]. Budding research since the onset of the pandemic in 2020 shows that in the wake of the COVID-19 pandemic, chatbots have a unique role [2]. When writing content for a chatbot in an acute emergency, focus can be given to remaining iterative and agile, leaning on complementary forces to triangulate data, and balancing global knowledge with a local focus.

The specificities of COVID-19 transmission have shown that individual action, when scaled to the community and beyond, can make a difference to the trajectory of a pandemic [10]. HealthBuddy+ is therefore a tool adapted to the individual, at a time when individual action has the possibility to slow the pace of the pandemic. It seeks to harness the role of the individual by providing answers to their diverse questions through practical and empowering messages on how to protect themselves and their communities while using a friendly approach and tone. In all, the receipt of targeted, simplified information in a relevant local language via an already used platform from trusted organizations has the potential to increase the resilience of individuals in an emergency.

The role of focal points has been crucial in enabling the adaptation of information sharing from regional level to country level based on their firsthand knowledge and understanding of language, culture, and context, particularly in the following: (1) supporting translation, given that the translation of scientific messaging is notoriously complex to get right; (2) understanding the local communication and information environments, identifying the most appropriate channels in which to embed the chatbot and the best approach to chatbot promotion; and (3) acting as bridges to the national authorities and key actors, and therefore the citizens.

Two years on from its launch, HealthBuddy+ continues to expand and adjust to meet emerging health emergency needs. Many lessons have been learned during the development and roll out of HealthBuddy+, with the most important being to remain both agile and innovative. In health emergencies, building and maintaining trust is paramount to support the response; to do so, it is vital to always keep individuals and at-risk communities at the center of response efforts. Building trust for HealthBuddy+ meant working closely with focal points in country offices and maintaining an open feedback loop so that HealthBuddy+ could always remain adaptable to local contexts.

As the chatbot technology and intelligence is in its infancy, there may remain gaps in functionality. However, these gaps also present an opportunity to learn what the bot cannot yet answer and therefore gain insight into what users want to know. In an unfolding emergency, risk communication must be localized to geographical location, language, and cultural context as much as possible without losing scientific rigor, which can be done through translating content into the local languages, dejargonizing technical terms, and harnessing user data to understand how local audiences communicate.

### Conclusions

Based on the experiences, challenges, and adaptations in the first 2 years of the HealthBuddy+ project, the WHO and UNICEF will continue to update and adapt the tool to meet the information-seeking needs of the public and support national authorities. Most notably, with the recent war in Ukraine and subsequent migration of millions of individuals to neighboring countries, HealthBuddy+ is being adapted to respond to this emergency. Working with focal points from Hungary, Poland, the Republic of Moldova, and Romania has helped this effort by consolidating crucial health information and resources all in one location for refugees on the move, illustrating the agility of HealthBuddy+ and what it is capable of. Additionally, with the unfortunate inevitability of future health emergencies, long-term funding must be mapped and secured to sustain such efforts and future fit-for-purpose tools. Additional research should also be carried out to understand the impact and sustainability of the project in nonacute emergency times.

## Abbreviations

RCCE: risk communication and community engagement
UNICEF: United Nations Children's Fund
WHO: World Health Organization
